# Primary Paediatric Bronchial Airway Epithelial Cell *in Vitro* Responses to Environmental Exposures

**DOI:** 10.3390/ijerph13040359

**Published:** 2016-03-24

**Authors:** Neil McInnes, Matthew Davidson, Alison Scaife, David Miller, Daniella Spiteri, Tom Engelhardt, Sean Semple, Graham Devereux, Garry Walsh, Steve Turner

**Affiliations:** 1 Child Health, University of Aberdeen, Aberdeen AB25 2ZG, UK; neilmcinnes@nhs.net (N.M.); m.davidson.09@aberdeen.ac.uk (M.D.); d.miller@nhs.net (D.M.); dmscornish@abdn.ac.uk (D.S.); sean.semple@abdn.ac.uk (S.S.); g.devereux@abdn.ac.uk (G.D.); 2 Institute of Medical Sciences, University of Aberdeen, Aberdeen AB25 2ZD, UK; a.r.scaife@abdn.ac.uk (A.S.); g.m.walsh@abdn.ac.uk (G.W.); 3 Department of Paediatric Anaesthesia, Royal Aberdeen Children’s Hospital, Aberdeen AB25 2ZG, UK; t.engelhardt@nhs.net

**Keywords:** children’s health, epithelial cell, environmental exposures, house dust mite, interleukin-8, lipopolysaccharide, tobacco smoke

## Abstract

The bronchial airway epithelial cell (BAEC) is the site for initial encounters between inhaled environmental factors and the lower respiratory system. Our hypothesis was that release of pro inflammatory interleukins (IL)-6 and IL-8 from primary BAEC cultured from children will be increased after *in vitro* exposure to common environmental factors. Primary BAEC were obtained from children undergoing clinically indicated routine general anaesthetic procedures. Cells were exposed to three different concentrations of lipopolysaccharide (LPS) or house dust mite allergen (HDM) or particulates extracted from side stream cigarette smoke (SSCS). BAEC were obtained from 24 children (mean age 7.0 years) and exposed to stimuli. Compared with the negative control, there was an increase in IL-6 and IL-8 release after exposure to HDM (*p* ≤ 0.001 for both comparisons). There was reduced IL-6 after higher compared to lower SSCS exposure (*p* = 0.023). There was no change in BAEC release of IL-6 or IL-8 after LPS exposure. BAEC from children are able to recognise and respond *in vitro* with enhanced pro inflammatory mediator secretion to some inhaled exposures.

## 1. Introduction

The airways are the initial site for encounters between inhaled exposures and the host immune response including macrophages and dendritic cells [1,2]. The airway epithelial cell is considered to have an important role in the regulation of respiratory inflammatory responses to inhaled foreign particles in adult and children [3,4]. Studying the response of children’s bronchial airway epithelial cells to environmental exposures is a challenge due to the inaccessibility of the paediatric lower airway.

Bronchial airway epithelial cells (BAEC) have been successfully collected from children by airway brushings of the trachea under general anaesthetic during clinically indicated interventions [5,6,7]. The role of the BAEC in the response of children’s airways to inhaled foreign matter is not understood but is important given the daily exposures to allergens, pollutants and microbial compounds experienced by children world-wide. What is currently understood about BAEC response to exposures such as second hand smoke (SHS), lipopolysaccharide (LPS) and house dust mite (HDM) comes from models using BAEC from animals or adults and immortalised cell lines. Studies in human lung epithelial cell lines have demonstrated that these exposures are generally associated with pro-inflammatory responses characterised by increased release of interleukins (IL)-6 and IL-8 [8,9,10] through pathways which include Nuclear Factor kappa-light-chain-enhancer of activated B cells (NFkB) and toll-like receptors [11,12,13].

There are no data which describe how BAEC in children respond to SHS, LPS of HDM and our previous work has demonstrated that BAEC in adults do not necessarily respond in the same way as in children [14,15]. In this study we collected and cultured BAEC from children and then exposed the cells to concentration ranges of SHS or LPS or HDM *in vitro*. Our hypothesis was that primary BAEC sampled from children will have increased pro inflammatory mediator release after *in vitro* exposure to common environmental factors. Based on our previous work [14,15,16,17], the mediators of primary interest were IL-6 and IL-8.

## 2. Materials and Methods

### 2.1. Recruitment

Children aged ≤16 years scheduled for routine ear nose and throat operations under anaesthesia were recruited between January 2011 and April 2013. Exclusion criteria included upper respiratory tract infection in the previous week and hereditary bleeding conditions. A lifetime history of asthma, eczema and hayfever, receipt of current asthma medications and exposure to parental smoking at home was obtained by a researcher-administered questionnaire. A bronchoscopy cytology brush (10-mm disposable cytology brush, BC 202D-2010, Olympus, Southend-on-Sea, Essex, UK) was used to obtain “blind” bronchial brushings to obtain airway epithelial cells. Ethical approval for the study was granted by the North of Scotland Medical Research Ethics Committee (approval numbers 09/S0802/122 and 12/NS/0107) and informed patient consent was obtained.

### 2.2. Second Hand Smoke

Side-stream second-hand tobacco smoke was generated by allowing a lit cigarette to burn while placed inside a 2 m^3^ exposure chamber. Respirable particulate matter was collected from the chamber air on to 37 mm polycarbonate filters placed inside a Higgins Dewell type cyclone with air drawn through it at 2.2 L/min by a Casella Apex pump [18]. Particulate matter was removed from the filters by sonication in distilled water (25 mL) for thirty minutes. After sonication the filters were left to soak overnight before being removed and dried. The particulate solution was then freeze dried, weighed and a stock solution of known concentration prepared. The particulate solution was added to growth media.

### 2.3. Cell Culture and Mediator Assay

Bronchial AEC were grown in submerged culture as previously described [15]. Subculture by trypsinization at 70%–90% confluence was undertaken twice and experiments performed on confluent cells at third passage in 12-well plates. The bronchial epithelial growth medium was supplemented with 1% fetal calf serum (FCS, Gibco, Life Technologies, Paisley, UK) as a source of essential toll-like receptor (TLR) 4 co-factors, particularly MD2 which is required for LPS response [19,20]. Tertiary confluent monolayers of BAEC were stimulated with media or IL-1β and tumour necrosis factor (TNF)α (both at 10 ng/mL, R & D, Abingdon, UK); or LPS (1, 10 and 100 µg/mL, Sigma-Aldrich Ltd., Poole, UK); or HDM (5, 25 and 50 µg/mL, Greer Laboratories, Lenoir, NC, USA. This contains 44 µg DerP1 and 625 EU per vial); and SSCS (5, 25 and 50 µg/mL). All exposures were performed at a final volume of 500 μL of media per well and were 24 h in duration. A semi-quantitative immunoarray using cytometric bead array analysis (CBA, Becton Dickinson, Oxford, UK) was performed on pooled samples from ten children. This informed the choice of a panel of cytokines and chemokines that were assayed in BAEC supernatants. The following mediators were measured: vascular endothelial growth factor (VEGF), regulated on activation normal T-cell expressed and secreted (RANTES), monocyte chemoattractant protein (MCP)-1, IL-3, IL-6, IL-8, IL-10, IL-17A, interferon (IFN)-γ, Granulocyte macrophage colony stimulating factor (GM-CSF), eotaxin, macrophage inhibitory proteins (MIP) 1-α and 1-β, intracellular adhesion molecule (ICAM)-1. Mediator release was determined in a negative control (*i.e.*, mediator alone for HDM and mediator plus FCS for LPS and SSCS exposures), a positive control (IL 1β and TNFα) and from culture supernatants from unstimulated and stimulated BAEC. All mediator results were normalised to cellular protein content of lysed monolayers quantified using the Bradford assay as previously described [15,16,17].

### 2.4. Analysis

Some result from the CBA were below the lower limit of detection (LLOD) and mediators were only considered for analysis if >75% of results were above the LLOD. The absolute difference in mediator concentration between negative control (media for HDM and 1% FCS for LPS and SSCS exposures) and supernatants from BAEC exposed to LPS, HDM and SSCS were compared using Friedman’s test since these data did not have a normal distribution. Our primary comparison was between negative control and exposure groups. We also compared differences within the three exposure groups as evidence of a dose-response relationship.

## 3. Results

### 3.1. Study Subjects

Bronchial AEC were cultured in 24 children, mean age 7.0 years (SD 3.1) including 14 boys. Three children had a history of asthma (all in receipt of asthma medications), six had a history of eczema, six had a hayfever history and three had ≥ one parent who smoked. Mediators meeting the inclusion criteria of >75% of CBA results above the LLOD were: IL-6, IL-8, VEGF, GCSF and ICAM. The results of the remaining mediators were not analysed. Stimulation with TNFα and IL1β resulted in the highest concentration of mediators compared to other exposures and the negative control (Table 1, Table 2, Table 3, Table 4, and Table 5).

### 3.2. Exposure to LPS

Compared to the negative control, there was no difference in any mediator release after exposure to LPS. Within the three exposure groups there were differences in VEGF and GM-CSF release, with the lowest concentrations of LPS were present after exposure to the intermediate concentration of LPS relative to the lowest and highest LPS concentrations (Table 3 and Table 4).

### 3.3. Exposure to HDM

**Figure 1:**
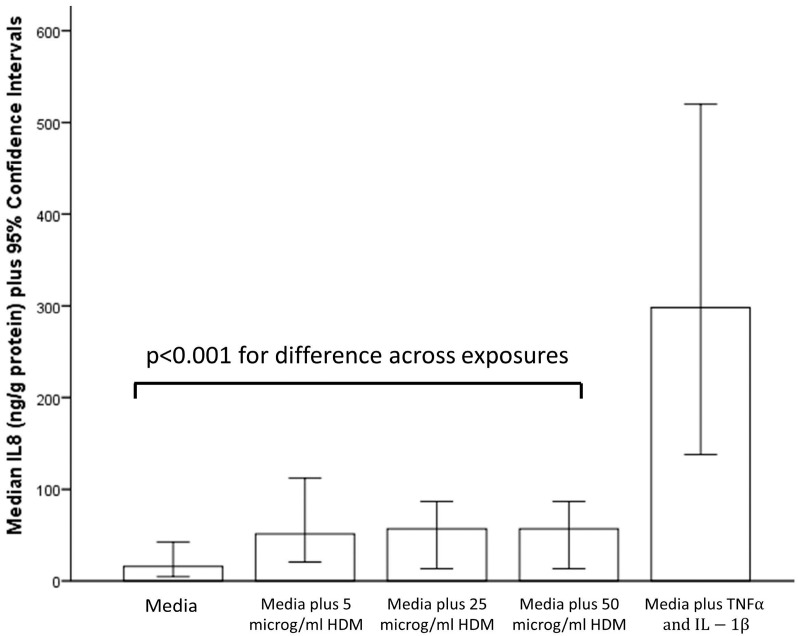
Interleukin-8 (IL-8) release from children’s bronchial epithelial cells after exposure to media alone (negative control), three different concentrations of house dust mite (HDM) and tumour necrosis factor alpha (TNFα) and interleukin-1 beta (IL-1β).

Compared to the negative control, concentrations of IL-6, IL-8 and ICAM but not GM-CSF and VEGF were elevated for all three HDM exposures (*p* < 0.001). There were no difference between concentrations of any mediators among the three exposure groups, Table 1, Table 2 and Table 5 and Figure 1.

### 3.4. Exposure to SSCS

There were no differences in mediator concentrations across exposure groups when the negative control was also included in the analysis. When only the three exposure groups were considered, concentrations of IL-6, GM-CSF and ICAM were greatest after the low exposure compared to the intermediate and high exposure (*p* < 0.005), Table 1, Table 4 and Table 5.

## 4. Discussion

This study addressed the question “how do isolated BAEC from children respond to common inhaled environmental exposures?” and the first notable finding was that exposure to house dust mite, at concentrations of ≥5 µg/mL, was associated with increased release of pro-inflammatory mediators. In contrast, there was no evidence of increased pro-inflammatory mediator release from BAEC following exposure to side stream cigarette smoke or lipopolysaccharide, even at the relatively higher concentrations of 50 and 100 µg/mL respectively. This proof-of-concept study demonstrates associations between release of pro-inflammatory cytokine release from BAEC in children following *in vitro* exposure to some common environmental exposures, and these findings support the paradigm that BAEC have a role in initiating the response of the innate immune system. Work is now required to explore the underlying regulatory mechanisms for mediator release in children with and without asthma.

Our findings are consistent with previous work from our group where we observed increased IL-8 release from neonatal nasal AEC after exposure to 5 µg/mL HDM and where there was no dose response for IL-8 release at concentrations higher than 5 µg/mL [16], suggesting that AEC are responding maximally to HDM at ≤5 µg/mL. Here we also replicate in BAEC our work in neonatal nasal AEC where release of IL-6 and ICAM, but not VEGF, was increased after exposure to HDM *in vitro* [17] and also the work by Rusznak *et al.* [10] who reported increased release of IL-8 and ICAM from adult BAEC after HDM exposure. The mechanism where HDM exposure leads to pro inflammatory mediator release from BAEC is not known. The LPS which might have contaminated our HDM extract might theoretically have caused the pro inflammatory response, but the lack of BAEC response to LPS in our study strongly suggests that the cytokine response to HDM is not explained by LPS co-exposure, although a synergistic effect cannot be ruled out. One potential mechanism for stimulating pro-inflammatory response from BAEC involves beta glucan moieties on HDM products [21] binding to TLR-2 [22]. Alternatively, or additionally, constituents of HDM extract can trigger intracellular calcium influx via an action on purinergic receptors [23] and by pathogen recognition receptors other than TLRs [24].

This was the first study to describe BAEC response to side stream cigarette smoke extract in any age group, and the results are partly consistent with the literature reporting effects of main stream CSE on BAEC in adults. Comer *et al.* [7] also report no increase in BAEC release of IL-6 using an *in vitro* model and observed increased IL-8 release relative to control at lower exposures but reduced release at higher exposure [8]. The apparent reduction in IL-8 response at higher exposure to cigarette smoke products seen in our study and elsewhere [8,25] might be explained by cytotoxicity at very high exposure concentrations. Cytotoxicity may also explain the lower BAEC production of GM-CSF and ICAM-1 after exposure to highest concentration of side stream smoke extract compared to the negative control. Witherden *et al.* [25] suggest that at very high exposures, CSE has an antioxidant effect on BAEC which suppresses a proinflammatory response. The response of BAEC to exposure to products of tobacco smoke is also dependent on the duration of exposure, since there is a diminishing IL-8 response to exposure to cigarette smoke between 20 min and 6 h [10]. An additional limitation of our model is that the particles within the side stream CSE will have settled onto the BAEC and result in prolonged direct contact between exposure and cell which does not occur *in vivo* due to mucocilliary clearance and activities such as change in posture and coughing. Cigarette smoke products might modify the structure of IL-8 and/or alter binding between IL-8 and the monoclonal antibody in the detection assay [10]. We speculate that we would have observed a pro-inflammatory BAEC response to lower concentrations of side stream cigarette smoke and for shorter duration.

The apparent absence of BAEC response to LPS in our study at the lowest concentration is consistent with many studies but there is evidence that higher LPS exposures can provoke a pro inflammatory response. One study of alveolar epithelial cells from adults reported IL-8 release was increased after exposure to 10 µg/mL LPS [26]. Studies in human alveolar cell line (A549) have also reported increased IL-6 and IL-8 release after exposure to LPS of 50 µg/mL [12] and 100 µg/mL [27] but not at lower exposures. Comer *et al* [8] report increased IL-8 but not IL-6 in adult primary BAEC after exposure to 25 µg/mL LPS. The difference between the present study and others [8,12,26,27] may be explained by known differences between: (i) alveolar and bronchial cell responses to LPS [27] (ii) results from cell lines and primary cell culture (iii) adults and children. The present results contrast with our recent work in neonatal nasal AEC where we observed increased release of IL-6, IL-8, GM-CSF and ICAM [17] after exposure to 100 µg/mL LPS. Differences between the present study and our work in neonates which might explain different outcomes for LPS exposure include the use of bronchial *versus* nasal cells, in the neonatal study fetal calf serum was not added to the culture and finally, age-related differences in responses to LPS may also occur.

One potential challenge to interpreting our results is determining the biologically relevance of the exposures. It is not valid to directly extrapolate the exposures applied in our *in vitro* experiment to those experienced by the airways *in vivo*. In one study, household dust had a geometric mean LPS concentration of 100 endotoxin units/mg [28] (range 4 to 2405 EU/mg) which is equivalent to between 2 and 50 ng/mg [29]. Assuming that 1 mL culture media weighs 1 g, our exposures of 1, 10 and 100 µg/mL are equivalent to 1, 10 and 100 ng/mg and thus approximately consistent with direct exposure of BAEC to average household dust.

There are strengths and limitations to the methodology used in the present study. One strength was using primary cells (and not cell lines) and this retains the phenotype of the individual, although there were insufficient individuals with asthma to allow a comparison of responses between children with and without asthma. A second strength is that *in vivo*, BAEC are exposed to LPS, HDM and SSCS held in airway surface liquid and our model replicates this situation, although *in vivo* the layer of airway surface liquid is much thinner that that used in our model. A limitation is that other cells which were not included in our model may modify BAEC response *in vivo*, e.g., dendritic cells, and while the study of inter cellular interactions is one method of extending our model, our focus for this study was BAEC response in isolation. A second limitation is that environmental exposures *in vivo* are usually intermittent whereas in our model the exposure was constant and for 24 h. A further limitation is that undifferentiated cells were exposed in our experiments and different results might have arisen if differentiated cells cultured at air-liquid interface had been used.

## 5. Conclusions

In summary, this is the first study to describe children’s BAEC responses to common inhaled environmental exposures, and we report that BAEC can respond to some exposures in the absence of other mediators of the immune/inflammatory response. A better understanding of the signalling mechanisms which regulate BAEC responses to HDM is now required.

## Figures and Tables

**Table 1:** Median and interquartile range (IQR) concentrations of interleukin (IL)-6 in supernatants from bronchial airway epithelial cell culture after exposure to lipopolysaccharide (L = lowest concentration = 1 µg/mL, I = intermediate concentration = 10 µg/mL and H = highest concentration = 100 µg/mL), house dust mite (L = lowest concentration = 5 µg/mL, I = intermediate concentration = 25 µg/mL and H = highest concentration = 50 µg/mL) and side stream cigarette smoke (concentrations the same as for house dust mite). Units are pg per mg protein content of the confluent cellular monolayer.

Exposure	Lipopolysaccharide Median (IQR) *n* = 23	House Dust Mite Median (IQR) *n* = 23	Side Stream Cigarette Smoke Median (IQR) *n* = 18
Negative control	5.8 (1.1, 15.0)	1.6 (0.1, 11.3) *	4.1 (0.8, 18.3)
Exposure to L	7.5 (1.5, 15.5)	7.4 (0.5, 33.3) *	7.4 (1.7, 22.1) ^‡^
Exposure to I	5.4 (0.9, 16.7)	13.5 (1.1, 22.8) *	5.1 (1.0, 16.0) ^‡^
Exposure to H	4.2 (0.6, 11.4)	9.8 (1.9, 23.9) *	4.9 (1.1, 10.9) ^‡^
Exposure to TNFa and IL-1B	64.1 (8.2, 132.8)	63.4 (8.2, 136.9)	57.4 (5.9, 79.9)

* *p* < 0.001 from Friedman’s test when three exposures groups plus negative control included; ^‡^ *p* = 0.023 from Friedman’s test when only the exposure groups were included.

**Table 2:** Median and interquartile range (IQR) concentrations of interleukin (IL)-8 in supernatants from bronchial airway epithelial cell culture after exposure to lipopolysaccharide (L = lowest concentration = 1 µg/mL, I = intermediate concentration = 10 µg/mL and H = highest concentration = 100 µg/mL), house dust mite (L = lowest concentration = 5 µg/mL, I = intermediate concentration = 25 µg/mL and H = highest concentration = 50 µg/mL) and side stream cigarette smoke (concentrations the same as for house dust mite).

Exposure	Lipopolysaccharide Median (IQR) *n* = 24	House Dust Mite Median (IQR) *n* = 22	Side Stream Cigarette Smoke Median (IQR) *n* = 18
Negative control	32.1 (18.1, 68.2)	18.5 (2.3, 46.1) *	34.1 (8.8, 78.0)
Exposure to L	28.5 (15.1, 105.7)	51.5 (15.1, 113.1) *	38.8 (18.4, 99.7)
Exposure to I	25.7 (12.5, 101.1)	56.3 (8.3, 121.2) *	34.0 (16.2, 104.7)
Exposure to H	35.7 (14.4, 84.0)	51.8 (12.0, 108.6) *	24.7 (14.7, 70.6)
Exposure to TNFa and IL-1B	323.8 (140.7, 552.7)	329.3 (138.0, 565.0)	263.9 (130.9, 373.0)

Units are pg per mg protein content of the confluent cellular monolayer. * *p* < 0.001 from Friedman’s test when three exposures groups plus negative control were included.

**Table 3:** Median and interquartile range (IQR) concentrations of vascular endothelial growth factor in supernatants from bronchial airway epithelial cell culture after exposure to lipopolysaccharide (L = lowest concentration = 1 µg/mL, I = intermediate concentration = 10 µg/mL and highest concentration = 100 µg/mL), house dust mite (L = lowest concentration = 5 µg/mL, I = intermediate concentration = 25 µg/mL and H = highest concentration = 50 µg/mL) and side stream cigarette smoke (concentrations the same as for house dust mite).

Exposure	LipopolysaccharideMedian (IQR) *n* = 24	House Dust MiteMedian (IQR) *n* = 23	Side Stream Cigarette Smoke Median (IQR) *n* = 18
Negative control	2.8 (1.8, 4.1)	3.0 (2.1, 4.6)	3.3 (2.4, 5.0)
Exposure to L	2.9 (2.4, 4.3) *	2.9 (1.7, 4.6)	3.6 (2.5, 4.8)
Exposure to I	1.8 (1.3, 2.9) *	2.7 (1.6, 5.1)	3.3 (1.8, 4.1)
Exposure to H	3.4 (1.7, 4.7) *	2.9 (1.9, 6.0)	3.4 (2.0, 4.6)
Exposure to TNFa and IL-1B	4.8 (1.9, 7.3)	4.8 (2.0, 7.4)	5.2 (2.3, 7.6)

Units are pg per mg protein content of the confluent cellular monolayer. * *p* = 0.008 for Friedman’s test across three exposure groups.

**Table 4:** Median and interquartile range [IQR] concentrations of granulocyte macrophage colony stimulating factor in supernatants from bronchial airway epithelial cell culture after exposure to lipopolysaccharide (L = lowest concentration = 1 µg/mL, I = intermediate concentration = 10 µg/mL and highest concentration = 100 µg/mL), house dust mite (L = lowest concentration = 5 µg/mL, I = intermediate concentration = 25 µg/mL and H = highest concentration = 50 µg/mL) and side stream cigarette smoke (concentrations the same as for house dust mite).

Exposure	Lipopolysaccharide Median (IQR) *n* = 24	House Dust Mite Median (IQR) *n* = 23	Side Stream Cigarette Smoke Median (IQR) *n* = 18
Negative control	0.06 (0.03, 0.33)	0.12 (0.00, 0.49)	0.05 (0.03, 0.32) ^‡^
Exposure to L	0.09 (0.01, 0.30) *	0.15 (0.02, 0.56)	0.11 (0.01, 0.38) ^‡^
Exposure to I	0.05 (0.00, 0.24) *	0.21 (0.03, 0.53)	0.03 (0.00, 0.24) ^‡^
Exposure to H	0.07 (0.03, 0.28) *	0.21 (0.00, 0.48)	0.02 (0.00, 0.30) ^‡^
Exposure to TNFa and IL-1B	0.38 (0.02, 2.04)	0.25 (0.02, 2.07)	0.68 (0.05, 1.99)

Units are pg per mg protein content of the confluent cellular monolayer. * *p* = 0.023 for Friedman’s test across three exposure groups; ^‡^ *p* = 0.019 for Friedman’s test across three exposure groups and *p* = 0.035 for Friedman’s test across three exposure groups plus negative control.

**Table 5:** Median and interquartile range (IQR) concentrations of intracellular adhesion molecule in supernatants from bronchial airway epithelial cell culture after exposure to lipopolysaccharide (L = lowest concentration = 1 µg/mL, I = intermediate concentration = 10 µg/mL and highest concentration = 100 µg/mL), house dust mite (L = lowest concentration = 5 µg/mL, I = intermediate concentration = 25 µg/mL and H = highest concentration = 50 µg/mL) and side stream cigarette smoke (concentrations the same as for house dust mite).

Exposure	Lipopolysaccharide Median (IQR) *n* = 24	House Dust Mite Median (IQR) *n* = 23	Side Stream Cigarette Smoke Median (IQR) *n* = 18
Negative control	0.58 (0.22, 1.23)	0.34 (0.00, 0.75) *	0.55 (0.21, 1.42) ^†^
Exposure to L	0.53 (0.28, 1.12)	0.71 (0.44, 1.46) *	0.89 (0.27, 1.76) ^†^
Exposure to I	0.30 (0.13, 1.11)	1.00 (0.27, 1.53) *	0.42 (0.07, 0.80) ^†^
Exposure to H	0.43 (0.21, 1.07)	0.78 (0.39, 1.60) *	0.31 (0.11, 1.70) ^†^
Exposure to TNFa and IL-1B	1.42 (0.46, 3.44)	1.58 (0.43, 3.73)	1.43 (0.60, 4.25)

Units are pg per mg protein content of the confluent cellular monolayer. * *p* = 0.001 from Friedman’s test when three exposure groups and negative control were included; ^†^ *p* = 0.031 for Friedman’s test across three exposure groups and *p* = 0.045 for Friedman’s test across three exposure groups plus negative control.
